# Supplementary material for: ProteinShader: illustrative rendering of macromolecules
Source: BMC Struct Biol. 2009 Mar 30;9:19. doi: 10.1186/1472-6807-9-19 (PMC2672931; doi:10.1186/1472-6807-9-19)
Supplement: Additional file 1 — ProteinShader program without source code. This compressed file contains the complete ProteinShader program including associated libraries, but no source code. A README.txt file gives an overview of the ProteinShader distribution, and the index.html file in the help subdirectory has directions on getting started with the program as well as a set of tutorials. [file 1472-6807-9-19-S1.zip › ProteinShader-beta-0_9_4-binary/help/api/org/proteinshader/gui/components/controlpanel/RadioPanel.Type.html]

RadioPanel.Type (ProteinShader API)


|  |  |  |
| --- | --- | --- |
| **Overview** \| **Package** \| **Class** \| **Use** \| **Tree** \| **Deprecated** \| **Index** \| **Help** | |  |
| **PREV CLASS**   **NEXT CLASS** | **FRAMES**    **NO FRAMES**     **All Classes** |  |
| SUMMARY: NESTED \| ENUM CONSTANTS \| FIELD \| METHOD | DETAIL: ENUM CONSTANTS \| FIELD \| METHOD |  |


---


## org.proteinshader.gui.components.controlpanel Enum RadioPanel.Type

```
java.lang.Object
  java.lang.Enum<RadioPanel.Type>
      org.proteinshader.gui.components.controlpanel.RadioPanel.Type
```

**All Implemented Interfaces:**: Serializable, Comparable<RadioPanel.Type>

**Enclosing class:**: RadioPanel

---

``` public static enum RadioPanel.Type extends Enum<RadioPanel.Type> ```

There are two slightly different
versions of the RadioPanel.

---

**Enum Constant Summary**

`ATOM`
  
          The ATOM version of the RadioPanel has a few extra options
in the 'Selected:' and 'Global:' menus, and has an extra
combo box menu with the label 'Apply To:' and menu choices
such as 'Entire AA', 'Backbone', 'Side Chain', 'CA',
*etc*.

`CARTOON`
  
          The Cartoon version is a little simpler than the ATOM
version.


| **Method Summary** | |
| --- | --- |
| `static RadioPanel.Type` | `valueOf(String name)`             Returns the enum constant of this type with the specified name. |
| `static RadioPanel.Type[]` | `values()`             Returns an array containing the constants of this enum type, in the order they're declared. |

**Methods inherited from class java.lang.Enum**

`clone, compareTo, equals, getDeclaringClass, hashCode, name, ordinal, toString, valueOf`

**Methods inherited from class java.lang.Object**

`finalize, getClass, notify, notifyAll, wait, wait, wait`

**Enum Constant Detail**

### ATOM

```
public static final RadioPanel.Type ATOM
```

:   The ATOM version of the RadioPanel has a few extra options
    in the 'Selected:' and 'Global:' menus, and has an extra
    combo box menu with the label 'Apply To:' and menu choices
    such as 'Entire AA', 'Backbone', 'Side Chain', 'CA',
    *etc*.

---


### CARTOON

```
public static final RadioPanel.Type CARTOON
```

:   The Cartoon version is a little simpler than the ATOM
    version.


**Method Detail**

### values

```
public static final RadioPanel.Type[] values()
```

:   Returns an array containing the constants of this enum type, in
    the order they're declared. This method may be used to iterate
    over the constants as follows:

    ```
    for(RadioPanel.Type c : RadioPanel.Type.values())
            System.out.println(c);
    ```

    :   **Returns:**: an array containing the constants of this enum type, in the order they're declared

---


### valueOf

```
public static RadioPanel.Type valueOf(String name)
```

:   Returns the enum constant of this type with the specified name.
    The string must match *exactly* an identifier used to declare an
    enum constant in this type. (Extraneous whitespace characters are
    not permitted.)

    :   **Parameters:**: `name` - the name of the enum constant to be returned. **Returns:**: the enum constant with the specified name **Throws:**: `IllegalArgumentException` - if this enum type has no constant with the specified name


---


|  |  |  |
| --- | --- | --- |
| **Overview** \| **Package** \| **Class** \| **Use** \| **Tree** \| **Deprecated** \| **Index** \| **Help** | |  |
| **PREV CLASS**   **NEXT CLASS** | **FRAMES**    **NO FRAMES**     **All Classes** |  |
| SUMMARY: NESTED \| ENUM CONSTANTS \| FIELD \| METHOD | DETAIL: ENUM CONSTANTS \| FIELD \| METHOD |  |


---

# *Copyright © 2007-2008*
